# Comparison of Antioxidant and Anti‐Melanogenic Activity of γ‐Oryzanol and Gallic Acid by Inhibiting Tyrosinase Activity in B16F10 Mouse Melanoma Cells

**DOI:** 10.1002/fsn3.70946

**Published:** 2025-10-11

**Authors:** Seyed Ahmad Emami, Elham Hadipour, Motahareh Boozari, Mahdieh Andalib, Maryam Asnaashari, Zahra Tayarani‐Najaran

**Affiliations:** ^1^ Department of Traditional Pharmacy School of Pharmacy, Mashhad University of Medical Sciences Mashhad Iran; ^2^ Department of Biology, Faculty of Sciences University of Guilan Rasht Iran; ^3^ Department of Pharmacognosy, School of Pharmacy Mashhad University of Medical Sciences Mashhad Iran; ^4^ Medical Toxicology Research Center Mashhad University of Medical Sciences Mashhad Iran; ^5^ Department of Animal Processing, Animal Science Research Institute of Iran (ASRI) Agricultural Research, Education and Extension Organization (AREEO) Karaj Iran; ^6^ Targeted Drug Delivery Research Center, Pharmaceutical Technology Institute Mashhad University of Medical Sciences Mashhad Iran

**Keywords:** γ‐oryzanol, gallic acid, MITF, rice bran, tyrosinase

## Abstract

Rice bran is abundant in gallic acid, and γ‐oryzanol comprises a mixture of ferulic acid esters and phytosterols. We compared the antioxidant and anti‐melanogenic activities of gallic acid and γ‐oryzanol to treat disorders caused by hyperpigmentation. The antioxidant activity was measured by 2,2′‐diphenyl‐1‐picrylhydrazyl radical (DPPH) and ferric‐reducing antioxidant power (FRAP) methods. Then, its effects on viability, reactive oxygen species (ROS) production, mushroom tyrosinase, and melanin content were investigated on B16F10 murine melanoma cell line. The antioxidant effects of γ‐oryzanol were higher than those of gallic acid in DPPH and FRAP tests. γ‐oryzanol and gallic acid did not show a significant cytotoxic effect and also reduced the amount of ROS. In addition, the reduction of mushroom tyrosinase activity in γ‐oryzanol was more than that in gallic acid. A decrease in melanin content was observed in γ‐oryzanol and gallic acid. So, it can be concluded that γ‐oryzanol has more antioxidant and higher inhibitory effects in the expression pathway of proteins involved in melanin synthesis in B16F10 cells than gallic acid.

## Graphical abstract

We compared the antioxidant and anti‐melanogenic activities of gallic acid and γ‐oryzanol to treat disorders caused by hyperpigmentation. The antioxidant activity measured by 2,2′‐diphenyl‐1‐picrylhydrazyl radical (DPPH) and ferric‐ reducing antioxidant power (FRAP) methods. Then, its effects on viability, reactive oxygen species (ROS) production, mushroom tyrosinase and melanin content investigated on B16F10 murine melanoma cell line.

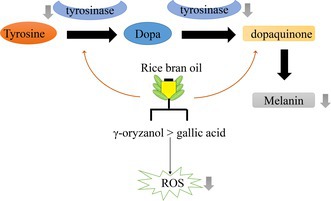

**Abbreviations**
AMVN: 2,2′‐azobis(2,4‐dimethylvaleronitrile)
BHA: butylated hydroxyanisole
BHT: butylated hydroxytoluene
CA: catalase
COX‐2: cyclooxygenase‐2
DCFH‐DA: 2′,7′‐dichlorofluorescein diacetate
DMSO: dimethyl sulfoxide
DPPH: 2,2′‐diphenyl‐1‐picrylhydrazyl radical
ERK1/2: extracellular signal‐regulated kinase 1 and 2
FBS: fetal bovine serum
FRAP: ferric reducing antioxidant power
GPx: glutathione peroxidase
GSH: glutathione reductase
H_2_O_2_
: hydrogen peroxide
HO‐1: heme oxygenase‐1
IL‐1β: interleukin‐1β
IL‐6: interleukin‐6
iNOS: inducible nitric oxide synthase
L‐DOPA: levodopa
MDA: malondialdehyde
MITF: microphthalmia‐associated transcription factor
NF‐Κb: nuclear factor kappa B
PPO: polyphenol oxide
PS: penicillin/streptomycin
ROS: reactive oxygen species
RPMI: Roswell park memorial institute medium
SOD: superoxide dismutase
TNF‐α: tumor necrosis factor alpha
TPTZ: 2,4,6‐tri(2‐pirydyl)‐s‐triazine
TRP‐1, 2: tyrosinase 1 and 2
UV: ultraviolet radiation

## 1 Introduction

As the largest organ of the body, the skin has the duty of protecting the internal body from the external environment with an excessive effect on the beauty of the body. In general, skin diseases comprise 34% of all diseases worldwide (Abbasi et al. 2010).

Tyrosinase is a copper‐containing polyphenol involved in melanogenesis, which is present in a large amount in mushrooms, higher plants, and animals (Yen et al. 2002). MITF and proteins related to tyrosinase 1 and 2 (TRP‐1, 2) help produce melanin (Yoon et al. 2013).

Melanin pigment is necessary to protect human skin from harmful rays. But excessive melanin production induces hyperpigmentation disorders such as melasma, sun spots, freckles, acne scar pigments, and senile lentigines (Hearing 2011; Tripathi et al. 1992).

One of the clinical applications of tyrosinase enzyme inhibitors is to prevent excessive accumulation of melanin pigment in mammals (Schallreuter et al. 2009). Several potent and moderate inhibitors of tyrosinase enzyme, from natural and synthetic sources such as arbutin, kojic acid, and hydroquinone, have been used in the last decade as bleaching or strong anti‐pigmentation agents because they can inhibit the production of melanin in the skin (Jimbow et al. 1974).

Phytosterols are potent antioxidants and control the growth, development, and maintenance of membrane fluidity in plants.

γ‐Oryzanol is one of the phytosterols derived from rice bran oil and consists of a mixture of plant sterols esterified to phenol and ferulic acid. γ‐Oryzanol was first isolated in Japan in the 1950s and used as a medication to treat anxiety, menopause symptoms, peptic ulcer, and gastritis (Tamagawa et al. 1992; Rahiman et al. 2018). Rice bran oil with a high concentration of γ‐oryzanol contains about 1% to 10 mg/g, and unripe rice bran oil contains 1.5% γ‐oryzanol. So, rice bran oil is the richest source of γ‐oryzanol, but this substance is also found in corn, barley, rye, wheat bran, and other edible oils. γ‐Oryzanol has anti‐inflammatory, antioxidant, and lipid‐lowering activity (Akaberi et al. 2021; Rahiman et al. 2018). It was shown that out of eight substances isolated from rice bran, γ‐oryzanol had a significant inhibitory effect on melanin synthesis (Jun et al. 2012).

Gallic acid (3,4,5 trihydroxybenzoic acid) is one of the most essential polyphenols in fruits, foods, and various plants such as oak, tea (green and black), sumac, grape seeds, apples, and rice bran oil (Wang et al. 2014; Fabian et al. 2010). Gallic acid prevents cellular damage by decreasing oxidative stress (Hsieh et al. 2017). By inhibiting the activity of tyrosine, gallic acid shows antioxidant, antibacterial, antiviral, antifungal, anticancer, anti‐inflammatory, and detoxification activities (Mansouri et al. 2013). Gallic acid can reduce the effects of oxidative stress in cells by increasing total thiol and the activity of the glutathione peroxidase (GPx) enzyme and reducing the amount of MDA (Mansouri et al. 2013). In addition, it has been approved as a food additive and is used widely in skin care products (Maurya et al. 2010). Besides, it has been shown that gallic acid has antioxidant properties (Kim 2007; Su et al. 2013).

In this research we have attempted to compare the antioxidant and anti‐melanogenic effects of γ‐oryzanol and gallic acid and investigate the underlying molecular mechanisms involved in melanin synthesis in the B16F10 mouse melanoma cell line.

## 2 Materials and Methods

### 2.1 Determination of Antioxidant Activity

#### 2.1.1 2,2′‐Diphenyl‐1‐Picrylhydrazyl Radical Assay

2,2′‐diphenyl‐1‐picrylhydrazyl radical (DPPH) radical scavenging activity was performed in a 96‐well microplate according to the protocol of Bajalan, Mohammadi with some modifications (Bajalan et al. 2016). 150 μL of various concentrations of γ‐oryzanol (4 mM) and gallic acid (4 mM) (Sigma, Germany) were added to DPPH radical solution (150 μL, 0.1 mM, Merck, Germany) in methanol and incubated for 30 min in the dark at room temperature. The absorbance of the samples was measured at 517 nm. Vitamin C was used as a positive control. The antioxidant capacity of the sample is expressed as DPPH radical scavenging activity (%):

$$\begin{array}{c} \text{DPPH radical scavenging activity}\;\left(\%\right)\\ =\left({\mathrm{Abs}}_{\text{control}}-{\mathrm{Abs}}_{\text{sample}}/{\mathrm{Abs}}_{\text{control}}\right)\times 100 \end{array}$$

Abs_control_ is the absorbance of DPPH radical in methanol and Abs_sample_ is absorbance of DPPH radical in sample/standard.

#### 2.1.2 Ferric Reducing Antioxidant Power Assay

Ferric reducing antioxidant potential was conducted according to Nemes, Szőllősi (Nemes et al. 2018). Ferric reducing antioxidant power (FRAP) reagent was prepared freshly before analysis. Then, 2,4,6‐tri(2‐pirydyl)‐s‐triazine (TPTZ) (5 mL, 10 mM) in HCl (40 mM) was mixed with FeCl_3_ (5 mL, 20 mM) and with acetate buffer (50 mL, 0.3 M, pH = 3.6). The 96‐well plates were then incubated at 37°C for 30 min before absorbance was recorded at 593 nm. Analysis of FRAP was performed by adding 20 μL of γ‐oryzanol and gallic acid to 180 μL of FRAP reagent. Results obtained for the samples were expressed as μmol Fe^2+^/l of extract.

### 2.2 Cell Culture and Treatment

B16F10 melanoma cells (Pasteur Institute, Iran) were cultured in Roswell Park Memorial Institute Medium (RPMI 1640; Sigma, Germany) with 10% fetal bovine serum (FBS; Gibco, USA) and penicillin and streptomycin (PS; Gibco, USA). They were then incubated at 37°C with 5% CO_2_ and 90% humidity. γ‐Oryzanol and gallic acid (4 mM) were dissolved in methanol to provide a stock solution.

### 2.3 Analysis of Cell Viability

First, 100 μL of suspension containing 10^4^ B16F10 cells was transferred to each 96‐well plate well. Then, cells were treated with concentrations of 0.001 to 100 μM of γ‐oryzanol and gallic acid for 24 and 48 h. After a day, 20 μL of resazurin dye (sigma, Germany) was added to each well. It was shaken for 10 min and incubated for 6 h until the blue color of the wells turned pink. Finally, the absorbance of samples at 600 and 570 nm was read in Synergy H4 Hybrid Multi‐Mode Microplate Reader (BioTek, Winooski, USA). Doxorubicin (1 mM) was used as a positive control (Hadipour et al. 2020).

### 2.4 ROS Generation

To determine the level of ROS generation, 10^4^ cells were treated with concentrations of 0.001 to 10 μM of γ‐oryzanol and gallic acid for 48 h. Then 100 μL of hydrogen peroxide (H_2_O_2_; Sigma, Germany) with a concentration of 24 mM was added to each well and samples were incubated for 30 min. Thereafter, 2′,7′‐dichlorofluorescein diacetate (DCFH‐DA; Sigma, Germany) (10 μL) added to each well and samples were incubated for 20 min. Finally, the fluorescent intensity of DCF was recorded with a Synergy H4 Hybrid Multi‐Mode Microplate Reader (BioTek, Winooski, USA) at the excitation wavelength of 485 nm and emission wavelength of 538 nm. Kojic acid (4 mM) was used as a positive control (Lim 1999).

### 2.5 Mushroom Tyrosinase Activity Assay

10^4^ Cells were treated with concentrations of 0.001 to 100 μM of γ‐Oryzanol and gallic acid for 48 h. Then, 160 μL L‐DOPA (5 mM) (Sigma, Germany) and 20 μL of mushroom tyrosinase (200 unit/mL) (Sigma, Germany) added to the cells and incubated for 30 min. Finally, the absorbance was compared at 475 nm by Synergy H4 Hybrid Multi‐Mode Microplate Reader (BioTek, Winooski, USA) (Daneshmand et al. 2022).

### 2.6 Melanin Content Assay

10^6^ cells treated with concentrations of 0.01 to 25 μM of γ‐oryzanol and gallic acid. After 48 h, cells were washed twice in *phosphate‐buffered saline* (PBS; Gibco, USA) then the melanin content was measured by adding 100 μL of NaOH (2 M) for 30 min at 100°C and its absorbance read at 405 nm (Daneshmand et al. 2022; Hosoi et al. 1985).

### 2.7 Molecular Docking

γ‐Oryzanol consists of three major components including 24‐methylene cycloartanyl ferulate, cycloartenyl ferulate, and campesteryl ferulate. To evaluate how the main ingredients of oryzanol interact with tyrosinase, a molecular docking analysis was performed using the Schrödinger software (Schrödinger LLC, New York). The three‐dimensional structure of mushroom tyrosinase bound to the inhibitor tropolone was sourced from the Protein Data Bank (PDB code 2Y9X). The selection of protein codes was based on prior in silico research (da Silva et al. 2017). The co‐crystallized ligand, all water molecules, and non‐interacting ions were removed from the receptor. Subsequently, side chain atoms and any missing hydrogen atoms were added. The next step involved calculating the Gasteiger charges for the system. Finally, the receptor underwent minimization and optimization using OPLS3. For ligand construction, these ligands were also optimized using the OPLS3 (Optimized Potential for Liquid Simulations) method. Final visualization was carried out with Chimera 1.8.1 (Pettersen et al. 2004).

### 2.8 Statistical Analysis

Data values and results expressed as mean ± SD of three independent experiments in triplicates, and Prism 8 software was used for statistical analysis of data and graphs. Comparisons between groups were performed using the Two‐way ANOVA statistical test and Sidak's multiple comparisons test, and *p* < 0.05 was considered a significant difference.

## 3 Results

### 3.1 Antioxidant Activity

#### 3.1.1 2,2′‐Diphenyl‐1‐Picrylhydrazyl Radical

The scavenging effect of γ‐oryzanol and gallic acid (0.01–100 μg/mL) was assayed compared with the concentration of 0.01 γ‐oryzanol and gallic acid (Table 1). As shown in the table, the antioxidant activity increases in a concentration‐dependent manner (*p* < 0.05) compared with the concentration of 0.01.

**TABLE 1:** DPPH radical scavenging assay (%).

Extracts (μM)	0.01	1	10	20	50	100
Gallic acid	10.16 ± 0.24^b^	41.69 ± 3.17^b^	58.19 ± 6.19^a^	73.15 ± 2.64^b^	83.15 ± 4.2^b^	87.1 ± 6.14^b^
γ‐Oryzanol	4.1 ± 0.03^c^	13.17 ± 1.82^c^	21.0 ± 3.64^b^	28.2 ± 1.56^c^	32.09 ± 5.01^c^	42.16 ± 0.1^c^
Vitamin C	21.04 ± 2.16^a^	35.15 ± 3.1^a^	62.94 ± 3.74^a^	79.3 ± 2.89^a^	97.64 ± 6.91^a^	98.7 ± 7.47^a^

*Note:* Each value represents as mean ± SD of triplicate experiments. Different letters in the column indicate significant differences (*p* < 0.05).

#### 3.1.2 Ferric Reducing Antioxidant Power

The Antioxidant activity of γ‐oryzanol and gallic acid (0.01–100 μg/mL) was assayed compared with the concentration of 0.01 γ‐oryzanol and gallic acid (Table 2). As shown in the table, the antioxidant activity increases in a concentration‐dependent manner (*p* < 0.05) compared with the concentration of 0.01.

**TABLE 2:** FRAP (μmol Fe^2+^/L).

Extracts (μM)	0.01	1	10	20	50	100
Gallic acid	19.75 ± 0.4^b^	41.02 ± 2.18^b^	58.13 ± 6.1^b^	71.9 ± 3.18^b^	136.04 ± 10.69^b^	295.16 ± 3.1^b^
γ‐Oryzanol	15.14 ± 2.1^c^	34.16 ± 2.1^c^	42.1 ± 3.18^c^	53.5 ± 4.6^c^	87.16 ± 4.9^c^	97.4 ± 6.92^c^
Vitamin C	55.16 ± 2.18^a^	91.15 ± 3.15^a^	153.12 ± 2.8^a^	201.15 ± 21.6^a^	280.1 ± 14.3^a^	315.05 ± 7.6^a^

*Note:* Each value represents as mean ± SD of triplicate experiments. Different letters in the column indicate significant differences (*p* < 0.05).

### 3.2 Comparison of Effects of γ‐Oryzanol and Gallic Acid on Cell Viability of B16F10 Cells

**FIGURE 1:**
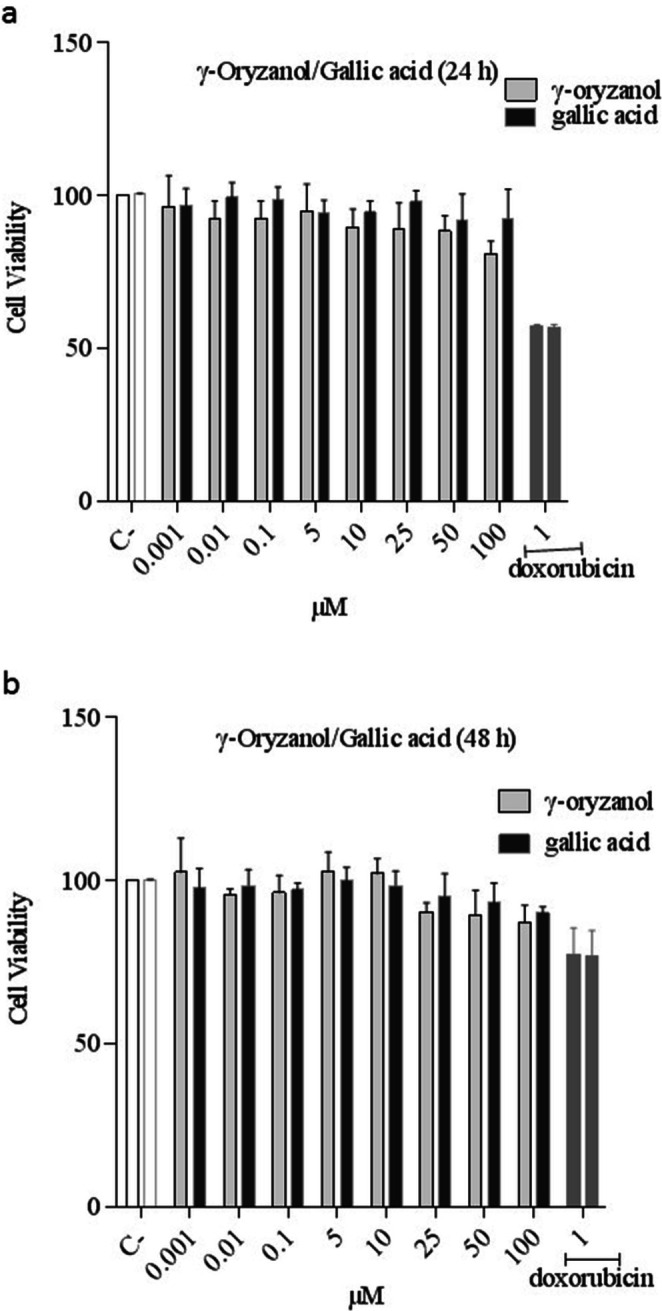
(a, b) Comparison of the effect of γ‐oryzanol and gallic acid on cell viability in B16F10 melanoma cells. The viability of B16F10 melanoma cells was evaluated after treatment with γ‐oryzanol (0.0.001–100 μM) and gallic acid (0.0.001–100 μM) for 24 and 48 h. Data are expressed as mean ± SD (*n* = 3) of three independent experiments. Doxorubicin (1 mM) was as positive control.

γ‐Oryzanol and gallic acid (0.001 to 100 μM) do not show cytotoxic effects compared to the control group after 24 and 48 h on B16F10 cells. Doxorubicin (1 mM) as a positive control reduces cell viability. For the following experiments, the cells were pretreated with different concentrations of γ‐oryzanol and gallic acid for 48 h as the optimal time (Figure 1a,b).

### 3.3 Comparison of Effects γ‐Oryzanol and Gallic Acid on B16F10 Cellular ROS Level

**FIGURE 2:**
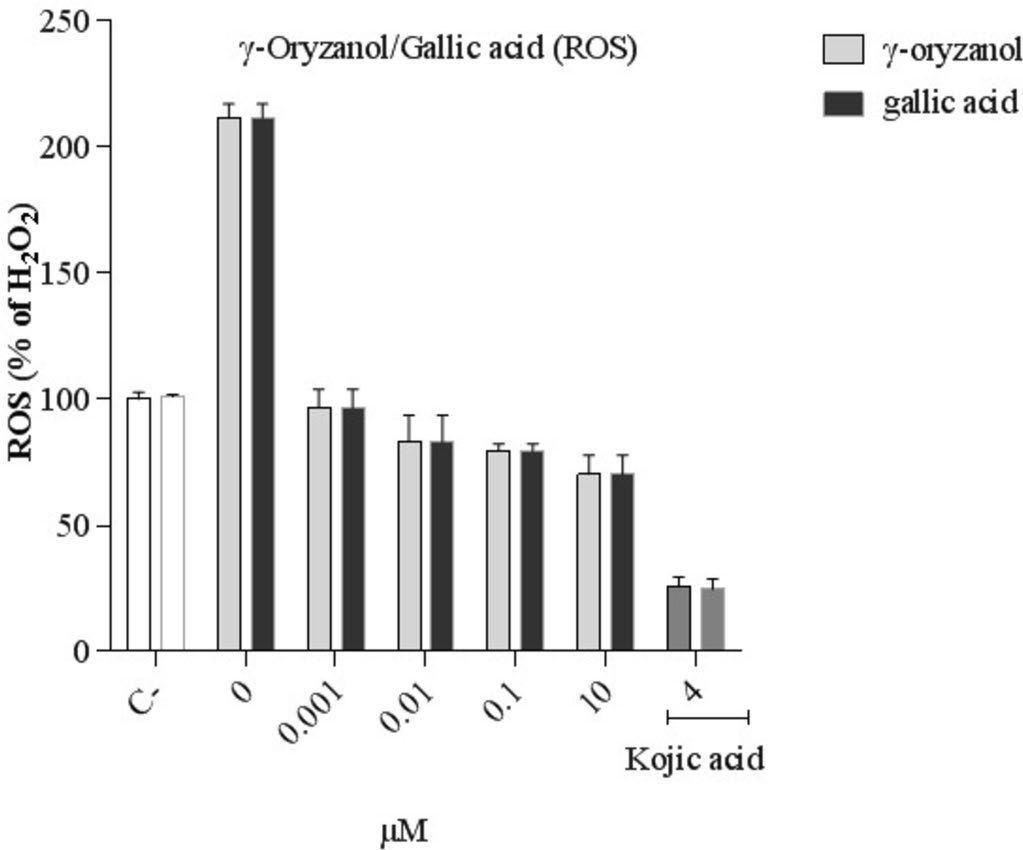
Comparison of the effect of γ‐oryzanol and gallic acid on ROS generation in B16F10 melanoma cells. The cells treated with γ‐oryzanol (0.001–10 μM), gallic acid (0.001–10 μM) and, Kojic acid (4 mM) for 48 h. Data are expressed as mean ± SD (*n* = 3) of three independent experiments.

As seen in Figure 2, treatment with H_2_O_2_ elevates the ROS content. However, treating cells with γ‐oryzanol and gallic acid reduces the ROS content in B16F10 cells induced by H_2_O_2_. Also, there is no significant difference between γ‐oryzanol and gallic acid. Kojic acid (4 mM) as a positive control, also reduces the ROS content (Figure 2).

### 3.4 Comparison of Effects of γ‐Oryzanol and Gallic Acid on Mushroom Tyrosinase Activity

**FIGURE 3:**
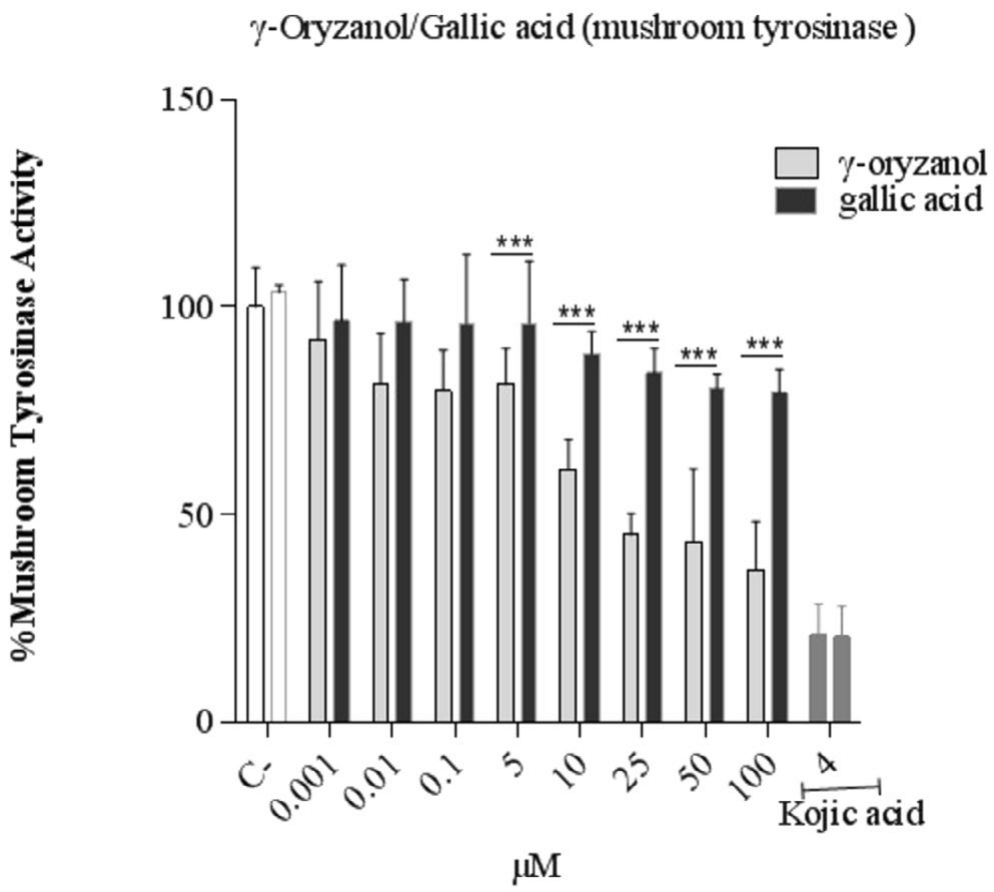
Comparison of the effect of the γ‐oryzanol on mushroom tyrosinase in B16F10 melanoma cells. The cells treated with γ‐oryzanol (0.001–100 μM), gallic acid (0.001–100 μM) and, Kojic acid (2 and 4 mM) for 48 h. Data are expressed as mean ± SD (*n* = 3) of three independent experiments. ***p < 0.001 compared between γ‐oryzanol and gallic acid.

Treatment of cells with γ‐oryzanol and gallic acid reduces the activity of mushroom tyrosinase. In comparison between γ‐oryzanol and gallic acid using two‐way ANOVA analysis, the reduction is significant in concentrations of 10, 25, 50, and 100 μM (*p* < 0.001) and γ‐oryzanol is more effective than gallic acid. Kojic acid (4 mM) as a positive control also has a decreasing effect on the activity of mushroom tyrosinase (*p* < 0.001) (Figure 3).

### 3.5 Comparison of Effects of γ‐Oryzanol and Gallic Acid on Melanin Production

**FIGURE 4:**
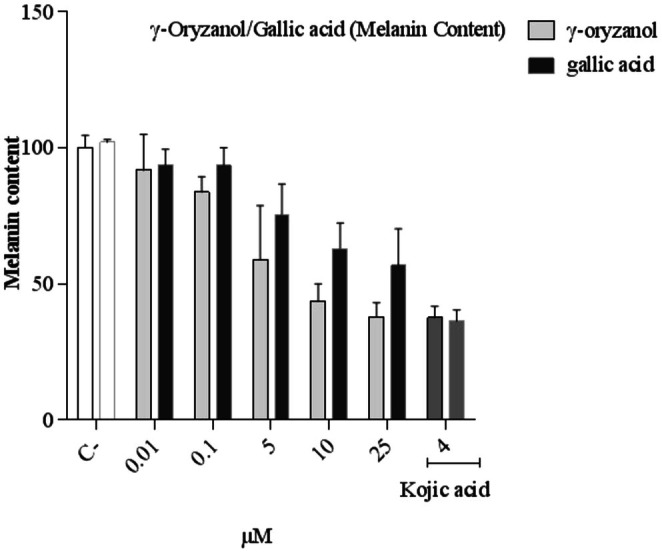
Comparison of the effect of the γ‐oryzanol and gallic acid on melanin content in B16F10 melanoma cells. B16F10 cells treated with γ‐oryzanol (0.01–25 μM), gallic acid (0.01–25 μM) and, Kojic acid (4 mM) for 48 h. Data are expressed as mean ± SD (*n* = 3) of three independent experiments.

Both γ‐oryzanol and gallic acid decrease melanin content with no significant difference between the two active compounds. Also, kojic acid (4 mM) as a positive control induces a decrease in melanin content (Figure 4).

### 3.6 Molecular Docking

**FIGURE 5:**
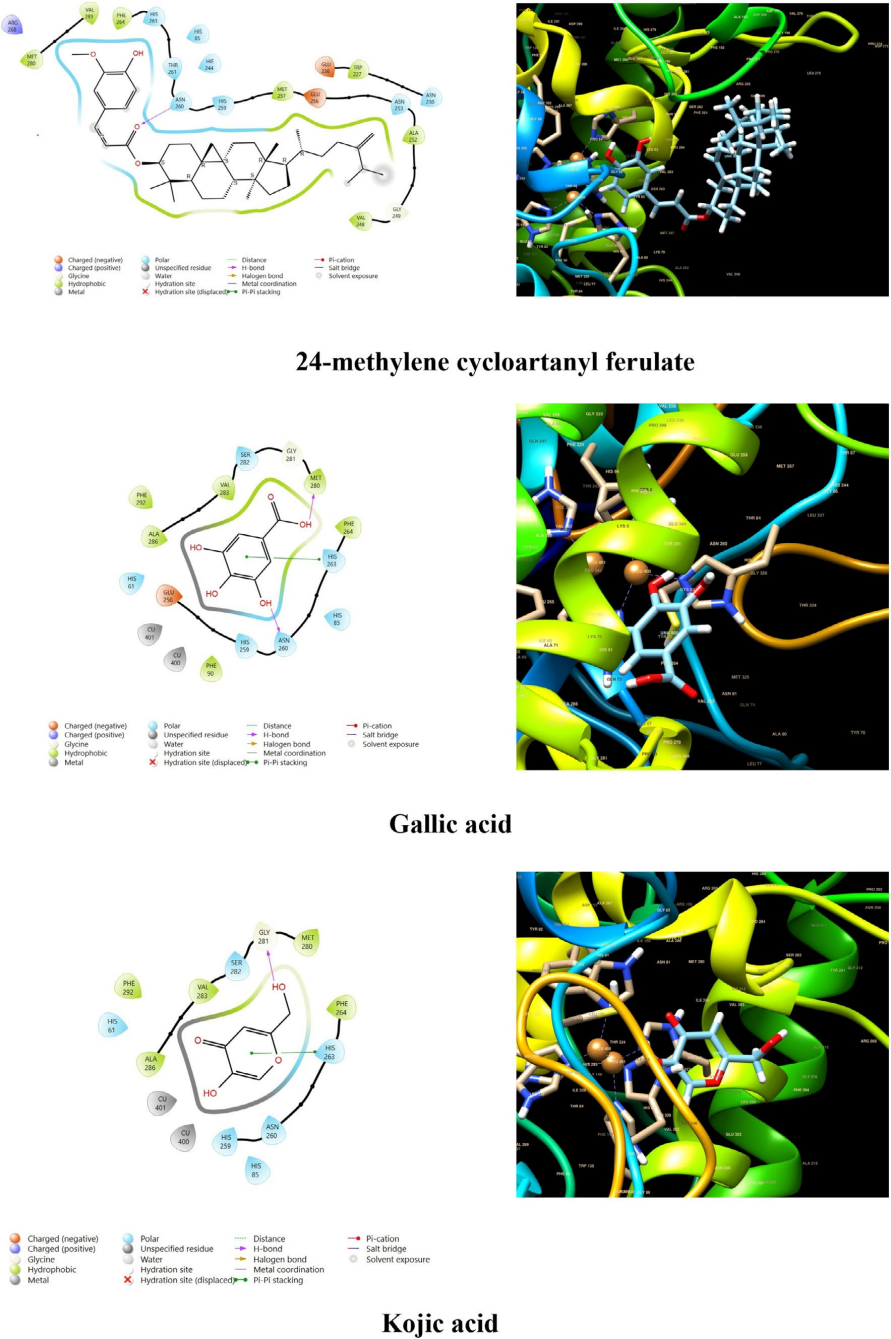
The compounds exhibiting the strongest tyrosinase inhibition and the lowest (most favorable) molecular docking scores. 24‐methylene cycloartanyl ferulate, Kojic acid and Gallic acid showed the best inhibitory effect against tyrosinase (Docking score: −5.206, −4.066 and −5.801 kCal/mol respectively).

**FIGURE 6:**
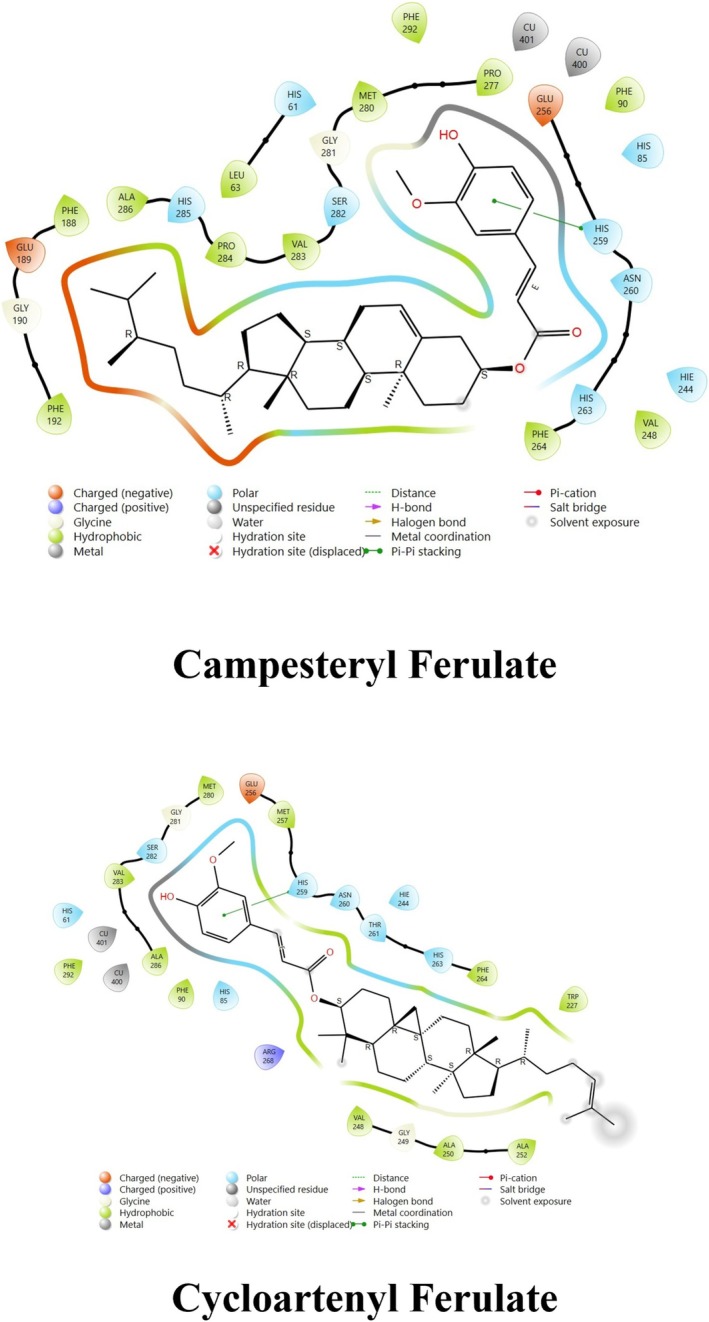
Binding interactions between the various ingredients of oryzanol and the enzyme tyrosinase.

The docking score of main ingredients of γ‐oryzanol against mushroom tyrosinase is shown in Table 3. The docked orientations revealed that all ligands were situated within the hydrophobic binding pocket encircling the binuclear copper active site. According to the molecular docking results, 24‐methylene cycloartanyl ferulate, Kojic acid, and Gallic acid showed the best inhibitory effect against tyrosinase (Docking score: −5.206, −4.066 and −5.801 kCal/mol respectively). The hydrogen bond between 24‐methylene cycloartanyl ferulate and gallic acid with Asn260 was important in inhibitory effect. Within the binding pocket, common interactions were established between all docked ligands and residues Asn260, His259, Gly281, Met280, and His263. 2D and 3D visualization of binding interactions between main ingredients of oryzanol against mushroom tyrosinase was shown in Figures 5 and 6.

**TABLE 3:** Docking Score for γ‐oryzanol ingredients, gallic acid and kojic acid against mushroom tyrosinase.

Compound	Structure	Docking score
24‐methylene cycloartanyl ferulate	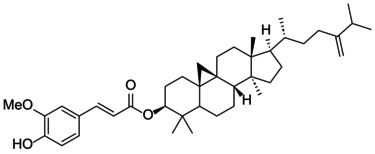	−5.206
Campesteryl ferulate	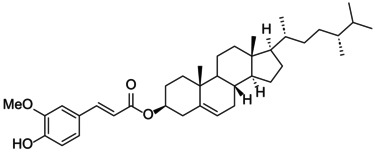	0.642
Cycloartenyl ferulate	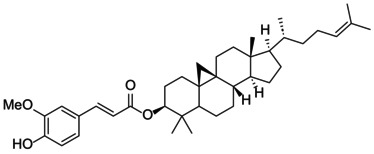	0.416
Kojic acid	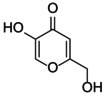	−4.066
Gallic acid	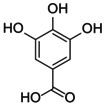	−5.801

## 4 Discussion

In the current study, we compared the antioxidant and anti‐melanogenic activity of γ‐oryzanol and gallic acid in B16F10 mouse melanoma cells. Our findings indicated that γ‐oryzanol has higher antioxidant and antimelanogenic effects than gallic acid on B16F10 cells.

Phenolic acids are phenolic compounds having one carboxylic acid group. Phenolic acids are divided into two sub‐groups: hydroxybenzoic and hydroxycinnamic acids. Hydroxybenzoic acids possess a common structure of C_6_–C_1_ and are derived from benzoic acid like gallic acid. Hydroxycinnamic acids have a C6–C_3_ skeleton. The four most common hydroxycinnamic acids are ferulic, caffeic, p‐coumaric, and sinapic acids. Typically, they are present in bound forms such as amides, esters, or glycosides and rarely in free form. γ‐Oryzanol comprises a mixture of ferulic acid esters and phytosterols (sterols and triterpenic alcohols). γ‐Oryzanol is thought to be a single compound, but as mentioned earlier, it is a mixture of ferulic acid esters and phytosterols (sterols and triterpenic alcohols). Unsaponifiable matter of crude rice bran oil contains high levels of components with antioxidant properties, including tocopherols/tocotrienols and γ‐oryzanol. Both gallic acid and γ‐Oryzanol, which are existing in rice bran, can be used as natural antioxidants for pharmaceutical purposes (Tamagawa et al. 1992; Wang et al. 2014; Fabian et al. 2010).

In previous studies, Xu et al. (2001) attempted to purify, identify, and evaluate the antioxidant chemicals in rice bran. The antioxidants in rice bran are tocopherols, tocotrienols, and γ‐oryzanol, which have shown remarkable antioxidant effects in the oxidation models of cholesterol and linoleic acid. Interestingly, γ‐oryzanol has more antioxidant activity in preventing cholesterol oxidation than tocopherols and tocotrienols (Xu et al. 2001). Saenjum et al. (2012) investigated the antioxidant and anti‐inflammatory effects of five varieties of Thai purple rice containing 1.23 to 9.14 w/w γ‐oryzanol. All extracts had acceptable antioxidant and anti‐inflammatory activity on murine macrophage cells (Saenjum et al. 2012). Ham et al. (2015) investigated the protective effect of rice bran unsaponifiable matters on oxidative damage of HepG2 cells induced by tert‐butyl hydroperoxide. The results showed that treatment with tert‐butyl hydroperoxide increases the activity of superoxide dismutase (SOD), catalase (CA), glutathione peroxidase (GPx), and glutathione reductase (GSH), while pretreatment with unsaponifiable matters of rice bran compounds effectively reduces oxidative damage (Ham et al. 2015) Juliano et al. (2005) compared the antioxidant activity of γ‐oryzanol and the synthetic antioxidants butylated hydroxyanisole (BHA) and reported relatively similar antioxidant effects. Therefore, it can be said that γ‐oryzanol prevents lipid peroxidation induced by *2,2′‐azobis (2,4‐dimethylvaleronitrile)* (AMVN) (Juliano et al. 2005). It has been shown that gallic acid in pistachio green hull can potentially protect triglycerides in soybean oil against peroxidation due to having an electron‐donating carboxylate anion (Delfanian et al. 2021).

To begin the study, the antioxidant activity of different concentrations of γ‐oryzanol and gallic acid was determined by free radical scavenging of DPPH and FRAP assay based on the electron transfer mechanism (Moreira et al. 2012). The free radical scavenging ability of antioxidants can be determined using stable free radicals like DPPH. The purple color of the DPPH radical disappears by abstracting a hydrogen atom from the antioxidant. As shown in Table 1, the DPPH radical scavenging effects of γ‐oryzanol are higher than gallic acid but still weaker than ascorbic acid. The radical‐scavenging activity of phenolic acids depends on the number of electron donor hydroxy and methoxy substitutions, which increase the stability of the phenoxy radicals. FRAP determines the reduction of TPTZ to a blue sample. The compounds with antioxidant activity can reduce Fe^3+^ to Fe^2+^. As shown in Table 1, the reducing capacities of γ‐oryzanol and gallic acid increased with the increase in concentration. The results of the DPPH and FRAP methods confirmed that γ‐oryzanol has a higher antioxidant activity than gallic acid and a good performance in free radical scavenging. The antioxidant activity of these compounds was mostly due to their redox properties as reducing agents, singlet oxygen scavengers, and hydrogen atom donors.

Our results also showed that treatment with different concentrations of γ‐oryzanol and gallic acid did not induce a significant reduction in cell viability at 24 and 48 h in melanoma B16F10 cells.

As mentioned, melanin plays an important role in the absorption of free radicals produced by the cell cytoplasm and UV rays of sunlight in the skin. So, substances with antioxidant properties can reduce the production of ROS and melanin (Brenner and Hearing 2008). Kojic acid is a natural compound derived from the fungi *Aspergillus*, *Acetobacter*, and *Penicillium* and is used as a lightening and anti‐stain agent in cosmetics formulation (Vashi and Kundu 2013). Its antioxidant activity is through the chelating of iron ions (Fe). Also, the inhibitory activity on melanin production is attributed to copper ion (Cu) chelation, which is present in the active site of the tyrosinase enzyme (Kahn 1995; Gonçalez et al. 2015). According to the study on the effect of γ‐oryzanol on human kidney stem cells in 2018, γ‐oryzanol significantly reduces the production of H_2_O_2_‐induced free radicals (Rungratanawanich et al. 2018). Also, pretreatment of human lymphoblast cells with ascorbic acid and gallic acid 30 min before the exposure to H_2_O_2_ induces a slight inhibition of DNA damage (Yen et al. 2002). In addition, pretreatment with fucoidan‐gallic acid increases cell viability and reduces ROS in pre‐osteoblast‐like cells induced by H_2_O_2_ toxicity (de Melo et al. 2022). According to the results, γ‐oryzanol and gallic acid both reduced the levels of oxygen free radicals significantly and similarly.

In this study, the activity of mushroom tyrosinase as an enzyme responsible for converting L‐DOPA to dopamine was investigated. The concentrations of 10, 25, 50, and 100 μM γ‐oryzanol and gallic acid significantly reduced the activity of mushroom tyrosinase. This decrease was greater in cells treated with γ‐oryzanol than in those treated with gallic acid. Considering that the basic structure of γ‐oryzanol is the aromatic core of ferulic acid, a study investigated the inhibitory effect of ferulic acid on mushroom tyrosinase, and it was shown that ferulic acid is an inhibitor of this enzyme (Gong et al. 2005). In addition, the structure of the phenol ring is important in showing the activity of anti‐tyrosinase, and the reason for the anti‐tyrosinase effect of gallic acid can be due to the presence of phenolic groups in its structure (Nerya et al. 2004; No et al. 2004).

Furthermore, γ‐oryzanol and gallic acid decrease the content of cellular melanin significantly in a similar manner.

Computational studies using molecular docking suggest that a key component of γ‐oryzanol (24‐methylene cycloartanyl ferulate) possesses a comparable ability to block the enzyme tyrosinase, which is involved in melanin production. This means the γ‐oryzanol component might be just as effective as gallic acid and kojic acid—known tyrosinase inhibitors—in preventing melanin formation. Therefore, it probably has a greater ability to deal with and treat damage caused by excessive melanin production than gallic acid.

By comparing the two compounds in rice bran, it can be concluded that both the antioxidant and anti‐melanogenic effects of γ‐oryzanol are higher than those of gallic acid.

## 5 Conclusions

According to the results of this study and by comparing the antioxidant and anti‐melanogenic effects between γ‐oryzanol and gallic acid, γ‐oryzanol showed higher reducing and inhibitory effects on the activity of mushroom tyrosinase. The presence of aromatic compounds called ferulic acid gives a higher effectiveness to γ‐oryzanol.

## Author Contributions

Mahdieh Andalib and Maryam Asnaashari collected samples and carried out experiments, analyzing the data. Elham Hadipour wrote the original draft and Seyed Ahmad Emami edited the final version of the manuscript. Zahra Tayarani‐Najaran supervised the project. All authors revised the paper critically for intellectual content and gave final approval of the version to be published.

## Conflicts of Interest

The authors declare no conflicts of interest.

## Data Availability

The data and material that support the findings of this study are available from the corresponding author upon reasonable request.
